# The GABBR1 locus and the G1465A variant is not associated with temporal lobe epilepsy preceded by febrile seizures

**DOI:** 10.1186/1471-2350-6-13

**Published:** 2005-03-30

**Authors:** Shaochun Ma, Bassel Abou-Khalil, James S Sutcliffe, Jonathan L Haines, Peter Hedera

**Affiliations:** 1 Department of Neurology, Vanderbilt University, 465 21st Avenue South, 6140 MRB III, Nashville, TN, 37232-8552, USA; 2 Center for Human Genetics Research, Vanderbilt University, 519 Light Hall, Nashville, TN, 37212, USA; 3 Department of Molecular Physiology and Biophysics, Vanderbilt University, 2215 Garland Ave, Nashville, TN, 37232, USA

## Abstract

**Background:**

Polymorphism G1465A in the GABBR1 gene has been suggested as a risk factor for non-lesional temporal lobe epilepsy (TLE); however, this genetic association study has not been independently replicated. We attempted to replicate this study in our cohort of patients with TLE. Furthermore, we also analyzed the coding sequence of this gene and searched for disease-causing mutations.

**Methods:**

We included 120 unrelated individuals with TLE that was preceded by febrile seizures (FS) who did not have any evidence of structural lesions suggesting secondary epilepsy. 66 individuals had positive family history of TLE epilepsy and 54 were sporadic. Each patient was genotyped for the presence of G1465A polymorphism. All exons of the GABBR1 gene were screened by single strand confirmation polymorphism method. Genotypes were compared with two independent matched control groups.

**Results:**

We detected two A alleles of the G1465A polymorphism in one homozygous control subject (0.87% of all alleles) and one A allele in a patient with TLE (0.45%, not significant). Other detected polymorphisms in coding regions had similar frequencies in epilepsy patients and control groups. No disease causing mutations in the GABBR1 gene were detected in patients with sporadic or familial TLE.

**Conclusion:**

Our results indicate that TLE preceded by FS is not associated with the polymorphisms or mutations in the GABBR1 gene, including the G1465A polymorphism. The proportion of TLE patients with FS in the original study, reporting this positive association, did not differ between allele A negative and positive cases. Thus, our failure to reproduce this result is likely applicable to all non-lesional TLE epilepsies.

## Background

Temporal lobe epilepsy (TLE) preceded by febrile seizures (FS) was historically considered to be an acquired disorder. However, recent studies have confirmed the existence of distinct genetic syndromes encompassing both FS and TLE [1,2]. Additionally, a positive history of FS in families with temporal lobe epilepsy is more common than expected by chance; this suggests that FS may be an initial manifestation of some temporal lobe epileptic syndromes [3-6]. The genetic etiology of TLE preceded by FS remains unknown. Two loci for this condition have been mapped, however, the causative genes have not been identified [1,2].

γ-aminobutyric acid (GABA) is the main inhibitory neurotransmitter system in the brain. Mutations in the GABA-A receptor subunit genes (GABR_A_), encoding products that form a heteropentameric chloride channel, have been identified as rare causes of idiopathic generalized epilepsies showing a Mendelian inheritance pattern [7]. However, mutations in the GABR_A_genes have thus far not appeared to be a major cause of TLE or other focal epilepsies [8].

The second, or B-subtype of GABA receptors (GABR_B_) function by acting through G-protein coupled signaling systems. Alteration in this GABA system during epileptogenesis is suggested from experimental data [9,10]. Furthermore, a previously described variant (G1465A) in the *GABBR1 *locus, encoding the GABR_B_β1 subunit has been reported in association with TLE, including TLE preceded by FS [11]. Genetic association studies are fraught with many difficulties and independent replication is important to establish the validity of any reported association [12]. We report genetic analysis of *GABBR1 *gene by thorough screening of *GABBR1 *exons for disease-causing mutations, and attempted replication of association at the G1465A polymorphism in a cohort of our patients exhibiting TLE preceded by FS.

## Methods

### Subjects

This study included 120 unrelated patients carrying a diagnosis of TLE preceded by FS. Informed consent, approved by the Institutional Review Board was obtained from every subject enrolled. A TLE diagnosis was made based on a history obtained from the patient and witnesses, with particular attention to descriptions of aura and complex partial seizure semiology, suggesting temporal lobe origin. Diagnoses were supported by electroencephalograph (EEG) recordings and MRI neuroimaging; some cases involved EEG-video recordings. MRI examination included 3D gradient echo imaging (Spoiled Gradient Recalled Acquisition) performed in the coronal plane with the slice thickness 1 mm, FLAIR and T2-weighted coronal sections; all coronal sections were perpendicular to the long axis of the hippocampus. Additional MRI sequences included T2-weighted axial and T1-weighted sagittal brain images. Only subjects with a reliable clinical classification were included in these studies.

Data collection involved age and duration at first FS, presence of any focal ictal or postictal features, total number of FS, and age at FS remission. Information was obtained from parents and/or older siblings. FS were classified as complex if they lasted longer than 15 minutes, had focal ictal or postictal features, or occurred more than once during the same day. Detailed family histories were obtained from all patients, including relatives with a history of epilepsy, FS, or both. Information was obtained from all first-degree relatives (mother, father, brothers, sisters, and children); information regarding second-degree relatives involved questions related to grandparents, grandchildren, nieces, nephews, aunts and uncles.

### Genetic analysis

DNA was isolated from peripheral blood from all subjects and relevant affected and unaffected family members enrolled in this study using standard methods. The current study focused solely on affected probands. The *GABBR1 *G1465A polymorphism was analyzed as previously described [13,14]. The PCR product was digested overnight with *EagI *restriction enzyme following the manufacturer's protocol (New England Biolabs; Beverly, MA). Products were analyzed by electrophoresis on 2% agarose gels and subsequent visualization of resolved fragments.

PCR primers flanking all exons were designed from published sequence and public genomic assemblies covering the locus (primer sequences are available upon request) [15]. Exon screening was performed using single strand confirmation polymorphism (SSCP) analysis; abnormal mobility shifts were sequenced.

All polymorphisms identified, including G1465A, were analyzed in two distinct ethnically- (Caucasians) and gender-matched control samples. The first comparison group consisted of DNA samples from 118 unrelated, healthy subjects obtained from the Vanderbilt Center for Human Genetics Research DNA Resource Core. The individuals from this group were absent any family history of epilepsy. The second control group corresponded to the Caucasian panel of 100 DNA samples from healthy individuals purchased from the Coriell Cell Repositories [16].

### Statistical analysis

Allele frequencies comparisons between patient and control groups was determined using a chi-square test. Mann-Whitney U test was used to compare demographic and clinical characteristics between patients with epilepsy and control subjects.

## Results

The 120 case subjects enrolled in this study included 49 men and 71 women (average age 34.8 ± 14.9 years) with a positive history of TLE, in which FS preceded the onset of epilepsy. Average age of TLE onset was 22.81 ± 10.08 years, with an average syndromic duration of 12.65 ± 8.12 years. Positive family history of epilepsy was reported in 66 individuals, while 54 were apparently sporadic cases. These two subgroups did not significantly differ in their demographic composition; however, familial cases had a significantly earlier age of epilepsy onset compared to sporadic cases (13.64 ± 8.23 and 27.44 ± 11.78, p < 0.01). All familial cases had at least one TLE affected relative in either previous or successive generations, consistent with vertical transmission of this trait. Medically refractory epilepsy was present in 23 patients (19%).

Genotype analysis of the G1465A variant detected a single A allele in one subject with TLE, who exhibited poor seizure control in spite of optimal clinical management. This patient had a positive family history of TLE and FS on one parent's side of the family. Genotype analysis of both parents indicated the A allele was present in the unaffected parent from the other side of the family, and thus did not segregate with disease. Two alleles of the same variant were detected in the control group in one control subject who was homozygous for this polymorphism. Allele frequencies for case and control groups corresponded to 0.45% or 0.87%, respectively; p = 0.988). Genotype accuracy for G1465A was confirmed by combined sequence and SSCP analyses that showed distinct and unambiguous mobility shift patterns for both heterozygous and homozygous states.

SSCP analysis of all coding exons did not reveal any apparent disease-causing mutations in *GABBR1*. We did, however, identify one previously described nonsynonymous variant (C59T) in exon 1, resulting in an alanine to valine substitution in one patient. This known single nucleotide polymorphism (SNP) is present in 1–3% of normal subjects, and it has been reported in a homozygous state in one affected individual [13]. Given the known normative frequency of the C59T SNP (rs#1805056), control samples were not screened. Several SNPs were detected in exon 11, which also contains the G1465A, variant, previously reported to be in exon 7 [11]. We detected three previously reported synonymous (silent) sequence changes – T1473C (rs#N/A), T1476C (rs#N/A), and T1485C (rs#29225) [14]. They appeared to be present on the same haplotype, based on segregation analysis of carrier relatives with this allele and constant SSCP mobility shift patterns. The frequency of this three-SNPs haplotype was determined to be 10.83% (26/240, including one homozygous individual); there was no difference between sporadic and familial cases. Analysis of controls (236 chromosomes) initially detected an allele infrequency of 4.23%, suggesting a significant trend (p < 0.05). Subsequent analysis of the second control sample identified a frequency very similar to the TLE patient sample (18/200, 9%, p = 0.63). Finally, we identified several novel intronic SNPs not predicted to alter splicing, and their frequency did not differ significantly between the TLE and control samples (data not shown).

## Discussion

The 1465A allele at *GABBR1 *was proposed as a potential risk factor for TLE, particularly in patients with medically refractory seizures [11]. We were unable to replicate this result, and no association of this allele with TLE was detected in our cohort of patients. Furthermore, systematic screening of all *GABBR1 *exons did not identify any obvious disease-related variants or novel SNPs or haplotypes significantly associated with the development of TLE.

The conflicting results between our study and that reported by Gambardella et al, [11] are unlikely due to clinical differences in the subject population or insufficient power. The sample sizes were similar (120 in our study and 141 in the original report [11]) in both studies. Our cohort had a history of antecedent FS, due to our ongoing effort to characterize this TLE subtype. The samples analyzed by Gambardella et al.[11] were mixed in this regard, and included patients with and without FS. Only 14.8% of their patients had a positive history of febrile convulsions, but the presence or absence of an FS history did not correlate with carrier status of the 1465A allele (16.7% with vs. 14.5% without 1465A) [11]. Our patient sample was more heavily loaded towards those with positive family history (55%) when compared with the study by Gambardella and colleagues [11] whose patient samples comprised of 36.2% of patients with a family history of epilepsy.

The control 1465A allele frequency in the current study was somewhat higher than in the previous study (0.87% vs. 0.26%, respectively), however, the difference between these two populations is not significant. This is consistent with other studies that found <1% frequency for the 1457A variant with an exception of one report, which identified 10% frequency of this allele in a cohort of 50 patients [11,13,14]. Our results support the conclusion that G1465A is a rare polymorphism. We also verified the presence of G1465A polymorphism by SSCP and sequencing and we had a complete agreement between different methods, arguing against incorrect genotyping as an explanation of our negative finding.

A systemic screening of all exons at the *GABBR1 *locus identified alleles at three known SNPs in exon 11 that also contains the 1465A variant, thus defining a unique haplotype [14]. These sequence changes are silent without any amino acid change in the protein. The comparison of the haplotypic frequency with our first control group suggested a potential increase, although a plausible functionality is currently unclear. The nonreplication in the second control group leaves the initial trend in doubt, and compels a conservative interpretation.

Spurious association that cannot be subsequently replicated can be caused by several factors [17]. Other than an inadequate sample size, selection bias is probably the most likely explanation of discrepancies between association studies. Unrecognized population stratification has been detected even in studies with otherwise proper design [18]. The problem of population stratification despite a careful selection is also demonstrated by the differences between the frequencies of the 1465A variant between our two control groups. It has been suggested that two control groups should be used in association studies, one population-based and one family based [19]. We did not have a family-based control group available but our conflicting data between two independent population-based control groups suggest that this approach is also useful in confirmation of true genetic association.

## Conclusion

Our data suggest that variants in *GABBR1 *are not significantly involved in the pathogenesis of TLE preceded by FS. The proportion of TLE patients with FS in the original study, which reported this positive association did not differ between allele A negative and positive cases, and thus our failure to reproduce this result is likely applicable to all non-lesional TLE epilepsies.

## Competing interests

The author(s) declare that they have no competing interests.

## Authors' contributions

SM carried out the molecular genetic studies, participated in the results interpretation, and helped to draft the manuscript. BA-K participated in the design of the study and its coordination, and helped to draft the manuscript. JSS participated in the design of the study and helped to draft the manuscript. JLH participated in the design of the study and helped to draft the manuscript. PH conceived of the study, participated in its design and coordination, performed statistical analysis, and drafted the manuscript. All authors read and approved the final manuscript.

## Pre-publication history

The pre-publication history for this paper can be accessed here:


